# Digitalization of management control in hospitals: an interventionist case study

**DOI:** 10.1108/JHOM-10-2025-0720

**Published:** 2026-09-03

**Authors:** Stefano Baraldi, Andrea Mariani, Federico Umberto Mion

**Affiliations:** Department of Economics and Business Management Sciences, Università Cattolica del Sacro Cuore, Milan, Italy; Center of Research and Studies in Health Care Management (CERISMAS), Milan, Italy; Regional Hospital of Lugano, Ente Ospedaliero Cantonale, Lugano, Switzerland

**Keywords:** Controller, Digital technologies, Digitalization, Digital innovation, Interventionist research, Management accountant

## Abstract

**Purpose:**

This article aims to contribute to the debate on the digitalization of management control and the evolving role of controllers in healthcare organizations. Specifically, it uncovers recurring patterns through which digitalization progressively reshapes control activities and indirectly transforms the controller's role.

**Design/methodology/approach:**

Drawing on literature on digitalization in management accounting and performance measurement in healthcare, the study presents longitudinal empirical evidence from an interventionist case study of a Swiss public hospital.

**Findings:**

Despite the growing interest in digital technologies, digitalization unfolded through a limited set of interconnected use cases rather than widespread technology adoption. The evidence shows that the digitalization of management control follows multiple, coexisting patterns that progressively reshape control activities, shift control from retrospective evaluation toward more real-time and forward-looking orientations, and, over time, indirectly transform the controller's role. These transformations carry direct implications for how healthcare organizations govern performance across clinical, operational, and financial domains.

**Research limitations/implications:**

The findings derive from a single interventionist case in a Swiss public hospital, which limits statistical generalizability. The interventionist design also introduces risks of prospective bias and narrative selectivity in participants' accounts.

**Practical implications:**

This research offers a framework to understand how digitalization reshapes management control through changes in control activities and practices and highlights the need for organizations to develop integrated technical, analytical, and relational competencies for controllers in digitally transforming environments.

**Originality/value:**

The study advances the literature by identifying a patterned and processual explanation of how digitalization reshapes management control activities over time and how role transformation emerges as an indirect outcome of these changes.

## 1. Introduction

In healthcare, management control (MC) systems have evolved considerably over recent decades, driven by increasing organizational complexity, resource shortage, regulatory pressures, and a growing demand for accountability and quality improvement. The push for digitalization has both ameliorated and exacerbated these issues: digital technologies can provide more timely, granular, and integrated performance information, which has served to expand the scope of traditional management control systems (MCS). At the same time, those technologies radically change the volume and diversity of data available for performance measurement (PM), which requires that organizations alter how they collect and analyze data for decision-making and control (Raghupati and Raghupati, 2014; Santiago de Mendonça *et al.*, 2025). In short, the digitalization of MC (Fähndrich and Pedell, 2025) stands to significantly improve the overall performance of healthcare organizations but also reshape the role of the controlling function in the process. As owners of MC processes, healthcare controllers are increasingly expected to move beyond their traditional “bean counter” role to become value-adding partners who actively design, implement, and leverage digital tools (Andreassen, 2020).

Beyond its practical relevance, the digitalization of MC is theoretically significant because it alters how performance information functions within organizations, facilitating the shift of the control from retrospective evaluation toward increasingly real-time and forward-looking coordination and decision-making. In complex professional settings such as healthcare, where performance information operates as a core governance infrastructure linking clinical, operational, and financial domains, these changes have far-reaching implications for how control is enacted and how organizational actors interact.

Despite these shifts, the literature still exhibits a limited understanding of the “Alchemy” that underlies MC digitalization and specifically how controllers can drive a successful integration of new technologies into existing systems. Indeed, several scholars have complained about the lack of empirical studies on the digitalization of MC (Fähndrich and Pedell, 2025; Möller *et al.*, 2020; Papiorek and Hiebl, 2024). Existing knowledge remains fragmented and mainly technology-centric, often focusing on individual tools or isolated practices, providing limited insight into how digitalization reshapes the concrete activities through which MC is enacted over time. Little is known about the mechanisms through which digital transformation unfolds, why it follows heterogeneous trajectories, and how it translates into evolving configurations of the controller's role. In particular, there is a shortage of research that captures the phenomenon from a simultaneously holistic (impact on the entire spectrum of MC processes), practical (impact on real use cases), and longitudinal (impact over an extended period of time) perspective.

Following this stream of literature, we argue that the digitalization of MC in healthcare (and other complex) organizations is not a linear, straightforward process. Instead, the digital transformation of MC involves varying levels of intensity, speed and scope, unfolding across multiple, distinct patterns. These patterns are not merely empirical regularities but generative configurations through which digitalization becomes consequential for control work, progressively reshaping how performance information is produced, integrated, and mobilized within organizational processes. As a result, healthcare controllers are challenged to re-design their role within the organization. The main objective of this exploratory study is to investigate how digital transformation influences MCS in healthcare, with a specific focus on the evolving role of controllers.

Based on an interventionist approach (Huxham and Vangen, 2003; Jönsson and Lukka, 2007), the study analyzes the digital transformation that occurred in the MCS of the Swiss multisite hospital, “Ente Ospedaliero Cantonale” (EOC), over the last three years. The Swiss healthcare system provides a particularly suitable setting for this investigation, as it combines universal coverage, strong cantonal autonomy, activity-based reimbursement, and ongoing reforms toward integrated and value-based care. These features generate a data-rich and coordination-intensive environment where performance information operates as a core governance mechanism, allowing digitalization to become consequential for the functioning of MC practices. As a large public multisite hospital operating in a regulated yet competitive environment, EOC represents a particularly informative setting to observe how digitalization reshapes MC across organizational levels, professional groups, and geographically distributed units. The interventionist approach was adopted due to its suitability for examining real-time change in dynamic and multifaceted environments, and for capturing organizational change as it unfolds in practice (Huxham and Vangen, 2003; Jönsson and Lukka, 2007). The collaboration with EOC allowed us to collect rich, contextualized data over an extended period; leverage the iterative, multi-staged cycle of action research; triangulate multiple data sources and observe the hospital's digital transformation process.

This study advances the literature by reconceptualizing digitalization in MC as a progressive reconfiguration of control activities rather than the adoption of discrete technologies. Specifically, we show that digitalization transforms MC by reshaping how performance information is generated, integrated, and mobilized across organizational processes, thereby altering the temporal orientation of control from retrospective to increasingly real-time and forward-looking. Through this activity-level transformation, digitalization produces selective and heterogeneous trajectories of change across control domains and triggers a co-evolution between evolving control practices and the professional role of controllers thereby offering a process-based explanation of how and why digitalization produces uneven and selective transformations across MC domains. In this sense, digitalization is conceptualized not as a purely technological shift but as a processual and patterned transformation of how control work is performed, explaining why digital transformation unfolds unevenly across domains and why its organizational consequences vary across contexts.

The paper is structured as follows: First, we present the literature review on the digitalization of MC and the evolving role of controllers in healthcare. Next, we detail the qualitative interventionist methodology. We then describe the empirical findings, focusing on the three core digital practices (i.e. use cases). We then develop the discussion and, finally, present the conclusions, where we summarize the contributions, outline the study's limitations, and suggest directions for future research.

## 2. Literature review

This paper draws from three complementary literature streams: the digitalization of MC, the changing role of controllers, and the evolution of MCS in healthcare.

For decades, technological advancements have been a continuous source of pressure on MC roles, practices and systems (Becker and Heinzelmann, 2017; Youssef and Mahama, 2021). In this vein, several studies have demonstrated that the implementation of Enterprise Resource Planning (ERP) systems and management accounting-related modules has markedly affected MC processes, including operational planning, performance measurement systems (PMS), and costing practices (Granlund and Malmi, 2002; Sànchez-Rodriguez and Spraakman, 2012) (see Appendix Table A1 for the full list of acronyms and definitions of the technologies mentioned in the paper). As a result, controllers have standardized and automated several routine activities, allowing them to move toward more managerial and IT tasks thus “hybridizing” their role (Caglio, 2003). The recent literature has focused on the advent of digital technologies (such as robotic process automation, blockchain, cloud computing, big data analytics, machine learning (ML), artificial intelligence (AI), etc.) and their use within the ongoing transformation process of MC tasks, instruments, organization, and behavioral aspects (Fähndrich, 2023; Rikhardsson and Yigitbasioglu, 2018).

Overall, these research findings affirm that the so-called “digitalization of MC” (Fähndrich and Pedell, 2025) offers a multitude of both opportunities and challenges. On the one hand, companies recognize the benefits of digitalization for their strategic planning and control processes (Truant *et al.*, 2021). Indeed, the standardization and automation of labor-intensive, routine-based operations in the controlling function has helped improve the efficiency and quality of conventional control processes (Cinquini *et al.*, 2025; Kokina and Blanchette, 2019; Korhonen *et al.*, 2021; Martins *et al.*, 2025). In addition, digitalization has expanded controllers' capacity to transform data into information, generate and communicate usable insights for decision-making, and provide a comprehensive view of operations (Bedford *et al.*, 2025; Bergmann *et al.*, 2020; Bhimani and Willcocks, 2014; Fähndrich, 2023; Fähndrich and Pedell, 2025; Fehrenbacher *et al.*, 2023; Karina *et al.*, 2026). On the other hand, digitalization entails several challenges. A digital shift in MC may lead to faster but potentially less accurate decisions (Quattrone, 2016), increase the need for superior governance of data and technologies (Rikhardsson and Yigitbasioglu, 2018), and go along with the risk of staff reduction or replacement (Bhimani and Willcocks, 2014). It can also give rise to paradoxes (e.g. reliability vs. timeliness, simplicity vs complexity) and even anxiety among professionals (Al-Htaybat and von Alberti-Alhtaybat, 2017; Firk *et al.*, 2024). Ultimately, controllers may be pressured to defend their relevance relative to other functional departments and ultimately have to adapt their roles to meet the challenges imposed by digitalization (Möller *et al.*, 2020).

Against that background, recent research has intensively investigated the changing organizational role of controllers (or management accountants) (Oesterreich *et al.*, 2019). Within this emerging body of literature, roles have been polarized around two overarching templates (Andreassen, 2020; van Slooten *et al.*, 2024): bean-counters and business partners. Where bean-counters (or watchdogs) primarily act as custodians of organizational resources and financial performance (Friedman and Lyne, 1997; Lambert and Sponem, 2012), business partners are actively involved in operational and strategic decision-making, strategy execution, and change processes (Byrne and Pierce, 2007; Jarvenpää, 2007; Wolf *et al.*, 2015). According to empirical findings, these roles rarely occur in their pure forms; instead, there is a continuum between the two “dichotomous” templates that results in hybrid roles (Burns and Baldvindottir, 2005; Rieg, 2018; Rieg *et al.*, 2023). Similarly, while several scholars have suggested that controllers are increasingly shifting toward a business-oriented role, research from diverse sectors and countries shows that “there is still much traditional scorekeeping work to be done by controllers” and that the new role of business partner “is still wishful thinking rather than visible reality” (Oesterreich *et al.*, 2019, pp. 3–15). Although technological developments have often been deemed a primary driver of role changes (Caglio, 2003), scholars have only begun to explore the relationship between highly impactful digital technologies and controllers' changing roles (Andreassen, 2020; Oesterreich *et al.*, 2019; Rikhardsson and Yigitbasioglu, 2018; van Slooten *et al.*, 2024).

The limited number of studies looking at how digitalization is shaping the work of controllers have produced useful, albeit contradictory evidence. Oesterreich *et al.* (2019) described three new roles for controllers (the bean-counter, the data scientist, the business partner) but found little evidence that controllers' daily activities are shifting toward less traditional tasks. Andreassen (2020) illustrated that the use of digital technology can create narrower, more specialized roles for controllers, allowing other internal groups to push against the boundaries of controllers' professional jurisdiction. Bedford *et al.* (2025) indicated that automation technologies can significantly impact traditional bean-counter tasks: For instance, controllers can use advanced analytics to expand the types of decision-making support that they offer to business units and their managers, thus expanding their role as business partners. Similarly, Rautiainen *et al.* (2024) found that the digital era is pushing controllers to adopt an increasingly fluid identity, as they move between traditional accounting responsibilities and emerging data-driven, analytical, and partnering roles. van Slooten *et al.* (2024) showed that controllers experience greater role ambiguity and conflict in the context of digitalization when they adopt the posture of a watchdog rather than that of a business partner. Consistent with these findings, Rieg *et al.* (2023) examined the expectations of role senders (i.e. employers) by analyzing a large sample of job advertisements. They found that controllers are rarely assigned a single, clearly defined role; instead, job descriptions typically reflect hybrid configurations, with the watchdog role predominating over both the business partner and the scorekeeper. Finally, Kokina *et al.* (2021) identified five distinct roles (identifier, analyzer, explainer, trainer, and sustainer) that accountants can play when adopting process automation. Although their study explicitly called for further research on how automation is transforming the controller's role, subsequent studies have only partially addressed this issue, leaving open questions about how and when these sub-roles actually emerge in practice.

There is little doubt that digital technologies have transformed virtually all industrial sectors (Bhimani, 2020), and healthcare is no exception. The diffusion of digital technologies in this domain, commonly referred to as Healthcare 4.0, has been widely documented across multiple application areas and is increasingly viewed as a key response to the complex challenges faced by healthcare organizations, including improving operational efficiency, enhancing quality of care, and creating value for multiple stakeholders such as patients, regulators, and commissioners (Ibrahim *et al.*, 2025; Kraus *et al.*, 2021; Raghupati and Raghupati, 2014; Trincanato and Vagnoni, 2024). Digital infrastructures also played a crucial role in supporting healthcare systems during the COVID-19 crisis, enabling new forms of coordination, monitoring, and data driven decision making (Kunz *et al.*, 2025; Santiago de Mendonça *et al.*, 2025).

In recent years, the evolution of PM and MCS has become increasingly central in healthcare organizations, where they operate as core governance infrastructures shaping coordination, accountability, and decision-making processes (Grossi *et al.*, 2020; Nuti *et al.*, 2018; De Domenico *et al.*, 2025). In knowledge intensive and professionalized settings such as healthcare, PMS and MCS are deeply intertwined, operating at the intersection of managerial and professional logics and influencing not only evaluation and monitoring but also organizational learning, behavioral regulation, and strategic alignment (Grossi *et al.*, 2020; Prenestini *et al.*, 2024). Performance information is therefore used not only to assess results but also to guide behavior, coordinate action, and legitimize organizational priorities, simultaneously supporting performance improvement, learning, and accountability.

A defining feature of PMS in healthcare is the multidimensional nature of performance. Healthcare organizations must simultaneously pursue financial sustainability, operational efficiency, quality of care, patient safety, and patient experience, requiring integrated measurement frameworks capable of capturing multiple and sometimes competing dimensions of value (Nuti *et al.*, 2018; de Melo *et al.*, 2024). Prior research shows that performance indicators shape organizational attention and behavior, influencing priorities, reputational dynamics, and improvement trajectories. However, empirical evidence highlights persistent challenges, including fragmented information architectures, limited integration across clinical, operational, and financial domains, and gaps between PM and managerial action (De Domenico *et al.*, 2025; Di Falco *et al.*, 2024; Noto *et al.*, 2025). In such professional environments, the effects of PMS depend not only on what is measured but also on how performance information is interpreted, mobilized, and embedded within decision making and coordination processes, reinforcing the view of PMS as socio technical systems shaped by the circulation and use of information across organizational levels and professional groups (Grossi *et al.*, 2020; Marrone and Hazelton, 2019).

Within this context, digital transformation has emerged as a key driver reshaping both PMS and MCS in healthcare. Digital and data driven technologies such as advanced analytics, AI, and integrated information systems enable more real time, integrated, and forward-looking performance management, while facilitating the integration of financial, operational, and clinical data within broader digital ecosystems (Mauro *et al.*, 2024; Spanò and Ginesti, 2022; Kakale and Pinelli, 2026). At the same time, the adoption and effects of these technologies remain contingent upon complex technological, organizational, and environmental conditions, resulting in heterogeneous and nonlinear transformation trajectories across organizations (Korpal *et al.*, 2026; Sony *et al.*, 2023; Tortorella *et al.*, 2020).

Importantly, digitalization represents not only a technological shift but also a transformation in how performance information is produced, accessed, and mobilized within organizations. The growing availability and visibility of performance data enables more structured and frequent performance monitoring, enhanced coordination, and the routinization of data driven decision processes. More advanced analytics further extend PMS toward predictive and anticipatory functions, supporting proactive management of risk, uncertainty, and resource allocation. Nevertheless, organizational, cultural, and informational barriers continue to constrain the effective use of PMS and MCS for decision making and performance improvement (Barros *et al.*, 2026; Horenberg *et al.*, 2020).

Despite these advances, many studies have examined specific technologies or isolated aspects of PM, fewer have investigated how bundles of digital technologies jointly reshape the architecture, integration, and functioning of PMS and MCS over time. Moreover, we still know little about how such shifts alter the temporal orientation of control work and the role expectations placed on controllers. Similarly, limited attention has been paid to how digitalization progressively transforms control practices and how these transformations shape the evolving role of organizational actors. Addressing this gap, the present study examines the digitalization of MC in healthcare as a processual and activity level transformation affecting the integration, use, and temporal orientation of PMS and MCS, rather than as a mere technology adoption phenomenon.

Despite the growing body of literature on digitalization in MC and healthcare performance systems, several important issues remain unresolved. First, although prior research widely acknowledges that digital technologies are transforming MC and PM, most studies focus on individual tools or isolated practices such as dashboards, analytics, or budgeting (Möller *et al.*, 2020; Fähndrich, 2023; Trincanato and Vagnoni, 2024; Ibrahim *et al.*, 2025). As a result, existing knowledge remains fragmented and provides limited understanding of how digitalization reshapes the concrete activities through which MC and PM are enacted over time. While recent studies call for a processual perspective that captures how digital transformation unfolds (Begkos *et al.*, 2024; Fähndrich and Pedell, 2025; van Slooten *et al.*, 2024), empirical evidence remains scarce and largely static, particularly in healthcare settings where performance information plays a central coordinating role (Mauro *et al.*, 2024; Buttigieg *et al.*, 2017).

Second, although the literature recognizes that digitalization is context dependent and shaped by multiple technological, organizational, and environmental drivers (Korhonen *et al.*, 2021; Oesterreich *et al.*, 2019; Bedford *et al.*, 2025; Rautiainen *et al.*, 2024), limited attention has been paid to how specific triggers generate distinct trajectories of transformation. Importantly, digitalization does not necessarily originate from deliberate strategic planning but often emerges from contingent pressures such as operational disruptions, cost constraints, or environmental shocks. Existing research suggests that digitalization unfolds unevenly across organizations (Begkos *et al.*, 2024; Kjekshus and Bygstad, 2024), yet the mechanisms through which such variation emerges and the ways in which different change configurations develop over time remain underexplored.

Third, although a growing body of literature examines the evolving role of controllers and hybrid professionals in the digital era (Andreassen, 2020; Oesterreich *et al.*, 2019; Rautiainen *et al.*, 2024; van Slooten *et al.*, 2024), the relationship between role transformation and the digital reconfiguration of MC activities remains insufficiently understood. Prior research rarely investigates how changes in operational control practices translate into shifts in professional roles, responsibilities, and identities over time, particularly within complex settings such as healthcare.

Accordingly, this study addresses the following research questions:

RQ1.
How does digitalization reshape MC and PM activities over time?

RQ2.
What triggers and generative mechanisms explain different trajectories of digitalization in MC activities?

RQ3.
How does the digital transformation of MC activities translate into evolving configurations of the controller's role?

## 3. Research methods

We designed an interventionist case study to fulfill this research's exploratory purpose. Following Lukka and Wouters (2022), this study can be positioned along the interventionist research continuum as a practice-driven study with theoretical ambition. Rather than testing a pre-defined theoretical framework, the research was primarily activated by concrete organizational problems and the theoretical contribution emerged inductively from the engagement with practice. In this sense, the study aligns with what Lukka and Wouters (2022) describe as interventionist research that starts from practical relevance and progressively develops analytical generalizability through the identification of recurring patterns and mechanisms. This positioning explains both the longitudinal and collaborative nature of the research design and the choice to let empirical patterns guide the theoretical framing, rather than imposing pre-established conceptual categories on the data. We actively cooperated with the Swiss multi-site hospital EOC, while the Head of Finance & Administration at Lugano Regional Hospital (LRH) co-authored this paper. To mitigate potential insider bias, the co-author did not take part in the coding of the empirical material. Although “the various streams of interventionist research are blurry” (Jönsson and Lukka, 2007, p. 377), this case study is an action research project where scholars worked with members of an organization to address issues of genuine concern to them (Eden and Huxham, 1996). The explicit intention was to produce changes in the host organization's work processes (Lukka and Wouters, 2022). The action research approach is fairly common in healthcare, where the principles of evidence-based practice are well rooted in organizational routines and culture (Bate, 2000).

Interventionist research is widely acknowledged as being highly effective for building theory from practice in complex situations (Eden and Huxham, 1996; Westbrook, 1995). Here, an engaged researcher has an opportunity to open the “black box” of the target organization and glean unexpected insights that enhance emerging theories and ensure their practical relevance (Huxham and Vangen, 2003; Jönsson and Lukka, 2007). As long as the researcher is perceived as a non-threatening outsider by field actors, they can collect rich and meaningful data on what people do and say, thereby capturing real-time change processes over an extended period.

In this qualitative case study, the authors investigated how MC processes at LRH were digitalized over an extended period. The study was designed to examine what drove change in MC and what digitalization patterns unfolded. EOC represents a particularly suitable context for this investigation because it is a large public multi-site hospital operating within an insurance-based healthcare system characterized by competition with private providers, while undergoing an ongoing digital transformation within the controlling function. This combination provides a rich setting to observe how MC practices evolve over time under conditions of organizational complexity and external pressure. Its structural complexity, central role of the controlling function in coordinating multiple sites, and data-intensive environment make it especially appropriate for examining how digitalization reshapes coordination, performance information flows, and the role of controllers in practice.

Specifically, we analyzed: (1) the technologies adopted within the controlling function to support digitalization; (2) the concrete use cases emerging from their adoption; and (3) the resulting changes in MC processes.

The collaboration with EOC was developed within a professional community hosted by the Center for Healthcare Management Studies of Catholic University. Since 2022, the community has primarily focused on the changing role of controllers and the digital transformation of the controlling function. Today, the community hosts a group of controllers from 22 healthcare organizations located in Italy and Switzerland. As an active member of the community, EOC closely collaborated with the interventionist researchers from February 2022 to September 2025 to support the digitalization of their existing MC processes. During this extended period, the researchers repeatedly benefited from the multi-stage cycle (Kock, 2004; Susman and Evered, 1978) that typically characterizes action research: diagnosing, action planning, action taking, evaluating, and specifying learning. The researchers served as experts throughout the research intervention, facilitating reflections on the processes and bringing systematic knowledge to the planning and execution of digitalization initiatives (Coghlan, 2004; Jönsson and Lukka, 2007).

The empirical data were collected and analyzed (Eden and Huxham, 1996) through a combination of methods, including in-depth interviews, discussion meetings, observations, examination of organizational documents, involvement in training events, and access to internal data and software applications (e.g. dashboards, performance reports). Overall, the researchers interacted with 20 EOC representatives from various departments (see Table 1). This range of data sources and types allowed the research team to gain multiple (at times, competing) views of the change process at EOC, which enabled an extensive triangulation in the data analysis. To further strengthen analytical rigor, data analysis was conducted independently by the two academic authors, who are not employees of the focal organization, thereby reducing the risk of organizational bias or conflicts of interest.

**Table 1:** Profile of the EOC representatives

#	Position	Department	Interview (Y/N; hours)
1	Head of Finance and Controlling	Lugano Regional Hospital	Y; 3.5
2	Controller	Lugano Regional Hospital	Y; 2.5
3	Head of Finance and Controlling	Bellinzona e Valli Regional Hospital	N
4	Head of Finance and Controlling	Mendrisio Regional Hospital	N
5	Head of Finance and Projects	Rehabilitation Clinic	N
6	Controller	Education and Research	N
7	Controller	Bellinzona e Valli Regional Hospital	Y; 1.0
8	Controller	Bellinzona e Valli Regional Hospital	N
9	Operations planner	Lugano Regional Hospital	Y; 1.0
10	Head of Finance and Administration	Finance and Controlling	Y; 1.0
11	Head of Business Intelligence	ICT Development	Y; 1.0
12	Head of Clinic	Lugano Regional Hospital	Y; 1.0
13	Medical Director	Institute of Clinical Neurosciences	Y; 1.5
14	Controller	Rehabilitation Clinic	N
15	General manager	Lugano Regional Hospital	Y; 1.0
16	Head of Finance and Controlling	Ente Ospedaliero Cantonale	Y; 0.5
17	Head of Finance and Controlling	Rehabilitation Clinic	Y; 0.5
18	HR analyst	Lugano Regional Hospital	Y; 0.5
19	Head of nurses	Institute of Clinical Neurosciences	Y; 0.5
20	Head of nurses	Anesthesiology Department, Lugano Regional Hospital	Y; 0.5

From February 2022 to September 2025, 27 meetings were held between the EOC informants and the interventionist researchers to share ideas, discuss research findings and best practices, plan change initiatives, monitor their implementation, review results, address emerging issues, and learn from lessons. Following each meeting, detailed notes (date, participants, topics, durations, documents, meeting notes) were produced to form the data set for the research. Table 2 provides a description of each meeting.

**Table 2:** Meetings with EOC representatives

#	Theme of meeting	Attendants	Time (m/y)	Hours
1	Assessment of the EOC's existing MCS	1, 2, 3, 4, 5	February 2022	3.0
2	Mapping of the EOC's budgeting process	1, 2, 3, 4, 5	February 2022	3.0
3	Digital technologies for healthcare MCSs	1, 2, 3, 4, 5	March 2022	3.5
4	New challenges for healthcare MCSs (value-based healthcare, ESG)	1, 2, 3, 4, 5	May 2022	3.0
5	The evolving role of healthcare controllers (business partnership)	1, 2, 3, 4, 5	May 2022	3.0
6	EOC data warehouse (structure, improvement areas)	1, 2, 3	October 2022	3.5
7	Digital tools to improve budgeting processes	1, 2, 3	November 2022	3.0
8	Digital tools to improve reporting processes	1, 2, 3	November 2022	3.5
9	Use of PMSs to support HR processes (performance evaluation)	1, 2, 3	March 2023	3.5
10	Use of PMSs to support HR processes (rewarding)	1, 2, 3	March 2023	3.0
11	Expanding the scope of PMSs with big data (new KPIs)	1, 3	May 2023	4.0
12	Expanding the scope of PMSs with big data (business intelligence and data visualization tools)	1, 3	May 2023	3.0
13	Digital technologies to be adopted within the EOC controlling function	1	October 2023	4.0
14	Emerging issues in the introduction of digital technologies in the controlling function	1	October 2023	3.0
15	Integrating data visualization tools with the balanced scorecard (performance report)	1, 2	November 2023	3.0
16	Integrating data visualization tools with the balanced scorecard (goal setting)	1, 2	November 2023	3.0
17	Cascading the balanced scorecard (KPIs for clinical departments)	1, 3	February 2024	2.0
18	Cascading the balanced scorecard (KPIs for research departments)	1, 3	February 2024	1.5
19	Possible applications of AI to control processes	1, 3	March 2024	3.0
20	Emerging issues and opportunities in the use of data visualization tools	1, 3	March 2024	4.0
21	Developing predictive analytics to support forecasting processes	1, 3	November 2024	4.0
22	Leveraging business intelligence tools for the development of research dashboards	1, 3, 6	November 2024	3.0
23	Evolution of the controller's role within the process of digital transformation	1, 7	February 2025	3.0
24	Using digital technologies for automating control processes (continuous performance improvement)	1, 6	March 2025	3.5
25	Using digital technologies for automating control processes (forecasting)	1, 6	March 2025	3.5
26	Developing the BI platform to integrate clinical outcome information into PMSs	1, 6, 7, 8	June 2025	3.0
27	Developing the BI platform to provide activity-based information	1, 6, 7, 8	June 2025	3.0

In addition, three group-feedback sessions and 14 one-to-one interviews were held between May and September 2025 for an ex-post analysis of the digitalization process. All sessions were fully recorded and transcribed. The list of interviewees is reported in Table 1. The empirical material was analyzed through thematic analysis (Braun and Clarke, 2013), following an iterative and inductive process. In the first step, the two academic authors independently coded the full dataset (comprising meeting notes, interview transcripts, and documentary sources) using an open coding approach. Codes captured four dimensions: (1) triggers and contextual pressures activating change; (2) changes in control activities and in the production, integration, and use of performance information; (3) enabling technologies and their specific functions; and (4) implications for the controller's role. In a second step, codes were clustered into broader themes through repeated comparison across data sources and between the two researchers, enabling the identification of recurring patterns. Disagreements in interpretation were resolved through discussion until consensus was reached. In a third step, the emerging patterns were systematically connected to the theoretical streams discussed in the literature review, allowing the analysis to move from empirical regularities to analytically generalizable configurations. The three use cases presented in Section 4 represent the output of this process and are synthesized in Table 4.

## 4. Digitalizing MC processes: empirical findings

This research examines the digitalization process that took place in a multi-site Swiss hospital from the beginning of 2022 to September 2025. This section provides the background of the research site and illustrates the three main use cases that resulted from the MC digitalizing processes.

### 4.1 Research site

Encompassing approximately 280 acute-care public and private providers, the Swiss hospital sector operates within a highly decentralized and resource-intensive healthcare system that combines universal access with strong cantonal autonomy. Since the introduction of SwissDRG (Diagnosis-Related Group) in 2012, inpatient care has been reimbursed through standardized flat-rate case tariffs. Conversely, outpatient services are funded almost exclusively through mandatory health insurance premiums. Switzerland is implementing further reforms to modernize its reimbursement frameworks: a key milestone is TARDOC and bundled payments, the new outpatient tariff system that will become effective in January 2026. Even more transformative is the gradual adoption of EFAS by 2032. Under EFAS, inpatient and outpatient services will be co-financed by cantons and insurers to promote the development of integrated care pathways and reduce incentives for providers to select patients according to their reimbursement regime. These shifts will significantly impact hospital finances. Moving from activity-based reimbursement and fragmented funding streams to bundled payments and uniform co-financing will require hospitals to optimize further resource allocation and strengthen their financial governance by improving coding accuracy, upgrading MCS, and developing accurate forecasts.

**Figure 1:**
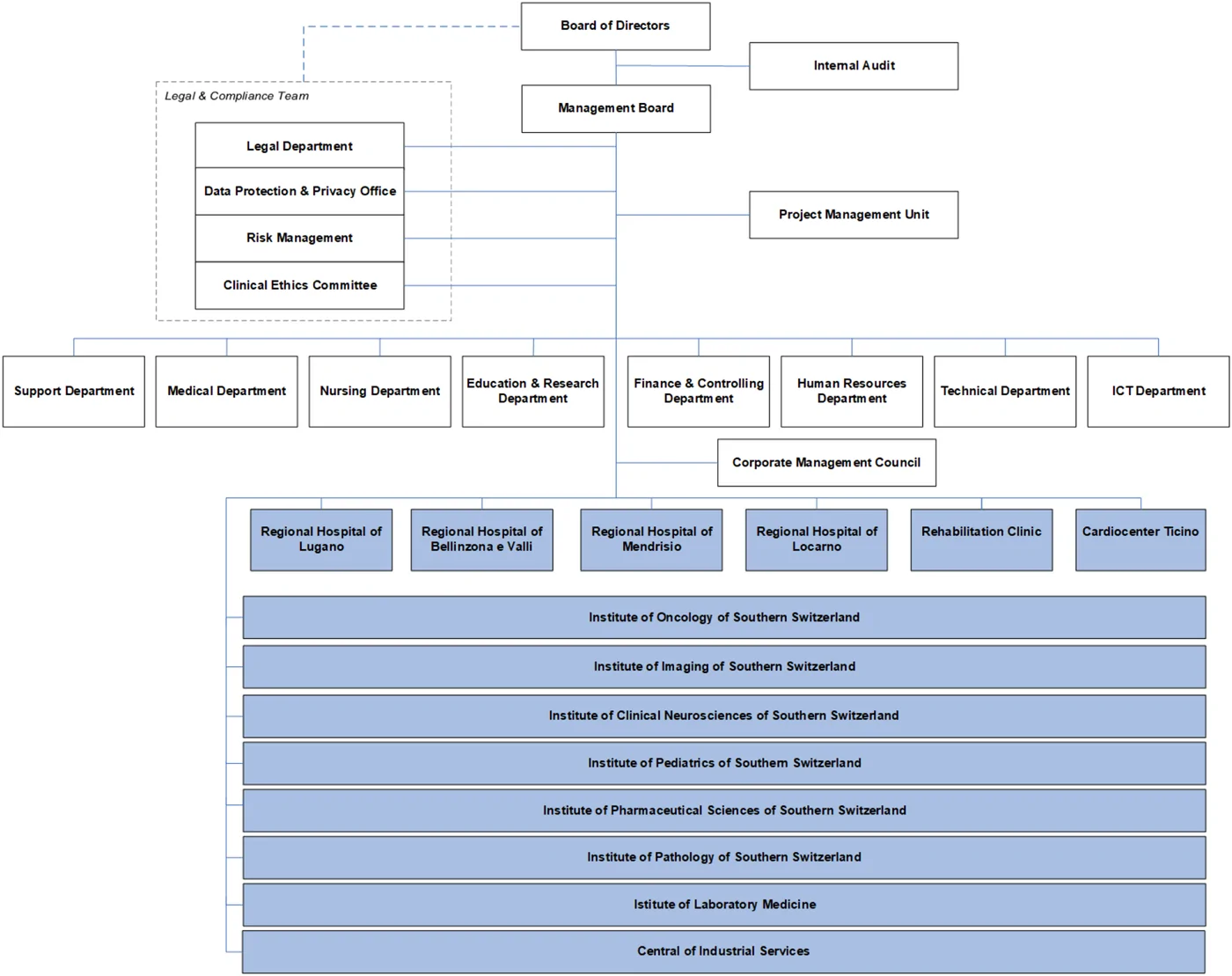
EOC organizational chart. Source: Adapted from EOC internal documentation

EOC is the primary public hospital network in the Canton of Ticino. Established in 1998 by cantonal law, EOC was created to consolidate and coordinate the provision of acute care across the region, ensuring equitable access to high-quality medical services. As a multi-site organization, EOC integrates four acute-care regional hospitals (Lugano, Bellinzona e Valli, Locarno, and Mendrisio) alongside several specialized institutes, such as the Institute of Oncology and the Neurocenter of Southern Switzerland. EOC's governance is grounded in public law, with strategic oversight exercised by a Board of Directors appointed by the Council of State of the Canton of Ticino. This board is responsible for defining the institutional strategy, approving budgets and annual accounts, and supervising management. Executive management is entrusted to the Management Board, led by the General Director and comprising directorates for the main corporate functions (e.g. medical affairs, nursing, finance, human resources etc.). In short, EOC is a matrix organization that combines centralized control with local operational autonomy. Figure 1 illustrates the governance structure and the positioning of the Finance & Controlling function within the overall organizational architecture.

Within this framework, the Finance & Controlling Department plays a pivotal role in EOC's financial stewardship, overseeing financial and control processes across all the institutes/regional hospitals. It includes specialized, corporate-level units such as Controlling, Financial Accounting, AP/AR, Coding, Tariffs, and Purchasing. On the other hand, each institute/regional hospital is supported by a Head of Finance & Administration (reporting hierarchically to the hospital Director and functionally to EOC's Chief Financial Officer) and a dedicated team of controllers. Within this structure, controllers act as boundary-spanning actors linking corporate-level financial governance with operational and clinical units. Their role combines budgeting, forecasting, performance monitoring, and support to decision-making at both the hospital and institute levels. While certain activities remain centralized (e.g. consolidation, tariff management, coding supervision), local controllers are directly involved in supporting clinical directors and hospital managers in interpreting performance data, identifying deviations, and coordinating corrective actions. This dual reporting and coordination structure makes the controlling function particularly sensitive to changes in information flows and digital infrastructures.

Since 2022, EOC has adopted three digital technologies that enabled the digitalization of MC processes at LRH:

1. ERP systems: SAP S/4HANA was adopted in 2023 and went live on January 2025; the full implementation of SAP Analytic Cloud is planned for 2026 to provide EOC with a single, integrated platform combining business intelligence (BI), predictive analytics, and planning functionalities.
2. Big data and analytics: EOC inherited a well-established platform for BI based on the XL Suite (XL Pharma, XL Net3, etc.); recently, however, the Information and Communication Technology (ICT) Development department strove to reduce the creeping fragmentation of software applications while incorporating a wider array of data sources.
3. Data visualization tools: Microsoft PowerBI Embedded was adopted in 2020 and is widely used throughout EOC; the migration to PowerBI Cloud is currently under evaluation.

No other digital technologies (e.g. blockchain, ML, AI, robotic process automation) have been introduced at EOC to reinforce and/or accelerate the MC digitalization.

### 4.2 Leveraging data visualization tools for creating strategic alignment

Like most Swiss hospitals during the COVID-19 pandemic, EOC was urged to implement an information platform that would optimize resource allocation processes on a daily basis and submit a report to authorities. The Controlling function developed an Excel report and gradually populated it with a wide range of data (e.g. bed/operating room occupancy rates, emergency room admissions, isolated patients). Over time, the growing burden of updating this platform and making its data accessible to more users encouraged EOC to invest in more sophisticated solutions. In the second half of 2020, the ICT Development department introduced new data visualization tools (Microsoft PowerBI) to make real-time data available to users. After a six-month transition, the new system was cascaded from corporate departments to local units (institutes, regional hospitals).

The LRH controlling team leapt at the chance to leverage the new system for challenging (and changing) a number of well-established practices in MC processes. Earlier, LRH's PM relied on a quarterly report (labeled a *Tableau de bord*) provided to the heads of clinical departments (e.g. general surgery, orthopedics and traumatology, urology, etc.). The report was produced in Excel, printed, emailed to users, and discussed in person (time permitting). Overall, the departmental income statement was reported to and analyzed by the MC unit, which reviewed detailed information on the various cost items, including both direct and indirect costs. Certain activity data (such as the patient volumes, the average length of stay, the case mix index, and the percentage of outlier cases) complemented this bulk of financial information. While intended for quarterly delivery, the report suffered significant processing delays and reached end-users (all internal stakeholders with managerial roles, e.g. clinicians, staff) roughly six weeks after the end of each quarter. Since the report was static, review meetings focused primarily on cost-allocation technicalities and accuracy, leaving little time to discuss the actual progress of clinical activities and outcomes, evaluating future trends and scenarios, and planning corrective actions.

Before COVID-19, performance information was provided mostly ex post. Regardless of its relevance, this information was communicated too late to the head of the clinical department, thus preventing them from closely monitoring performance, identifying possible issues, and tackling them through timely corrective actions. [EOC representative #13]

Following the adoption of data visualization tools, the LRH controlling team improved their digital skills by attending an intensive training program and eventually got recognized as PowerBI “superusers”. Since 2021, LRH controllers have made extensive use of this platform and implemented new performance dashboards that “broke with the tradition” of existing control practices. Specifically, performance information is now:

1. accessible to a broader variety of end-users, including nurse coordinators, in addition to the heads of clinical departments;
2. presented in a single, meaningful and intuitive view; visual representations (such as graphs, charts, maps, heatmaps, color-coding) systematically replaced table formats;
3. based not only on financial, but a multi-dimensional set of metrics, including patient safety and experience, clinical outcomes, productivity, etc.;
4. readily available (as much as possible, in real-time);
5. compared with targets and/or with internal (other departments within EOC) and external (other hospitals in Switzerland) benchmarks;
6. actionable for users who have information at hand and “navigate” through performance data looking for the possible root causes of poor performance and weak signals of future trends, taking proactive decisions and receiving prompt feedback about the effectiveness of their interventions.

In this context, the digitalization of MC practices followed an iterative process, characterized by trial and error. For example, the team initially adopted a bottom-up approach for the performance dashboards, asking the end-users to specify their informational needs and then designing their dashboards accordingly. While this was helpful for promoting a data-driven culture, this “customized” approach led to burdensome proliferation of PowerBI dashboards, which often prevented users from seeing a consistent picture of organizational and departmental performance.

The increasing number of dashboards and data sources sometimes makes it difficult for us to focus on what really matters, as too much information can easily become overwhelming in daily clinical practice. [EOC representative #19]

This tension was compounded by a deeper interpretive challenge. As the number of dashboards grew, users with stronger analytical backgrounds found it difficult to navigate precisely because the logic behind each visualization was not always transparent. Controllers from other EOC sites reported similar difficulties.

I find them very hard to use because you never really know what reasoning is behind them. If I didn't build it myself, it becomes almost impossible to interpret. [EOC representative #7].

Conversely, users with less analytical background tended to engage with dashboards more superficially, extracting headline figures without interrogating the underlying data. The result was an uneven appropriation of the technology across the organization, with the risk that data visibility did not automatically translate into data literacy.

Thanks to the extensive use of data visualization tools, the LRH controlling team radically increased the efficiency of the existing control processes (e.g. the collection, update, and calculation of the financial data included in the so-called *Tableaux de bord*), which encouraged a digitalization pattern aimed at creating strategic alignment toward organizational goals. Recognized as high-quality performance information providers, LRH controllers are now expected to foster data “democratization” and increase the visibility (as well as the accountability) of organizational goals “from bedside to board” (Buttigieg *et al.*, 2017) across hospital levels/areas. Expectedly, this evolutionary path required LRH controllers to develop new digital skills, broaden their knowledge of operational processes, and establish a trusted relationship with information users.

In the past, a cultural “divide” separated the clinical and administrative staff as two worlds apart. The development of performance dashboards gradually urged us to get into the good habit of fostering dialogue and communication among us, working more closely, and proactively collaborating for performance improvement. [EOC representative #13]

To reinforce this digitalization pattern, the LRH controlling team is currently planning to further invest in data visualization tools (PowerBI Cloud), increase the variety of performance data while enhancing their level of security, and refer to the corporate balanced scorecard (BSC) implemented to make performance information more salient and to cascade performance dashboards throughout the organization. More broadly, this transformation did not simply introduce new digital tools but progressively reshaped how PM activities were performed, increasing their timeliness, accessibility, and use for coordination and decision-making.

### 4.3 Leveraging business intelligence and analytics for automating operational review meetings

At the peak of COVID-19, the hospital introduced a structured set of operational meetings to address issues that needed immediate attention (e.g. access to intensive care unit, bed occupancy). Held daily at various organizational levels, these meetings were aimed at reviewing operational data and providing opportunities for problem-solving and continuous learning. The ICT Development department and the LRH controlling team joined forces to provide the meetings with valid and timely operational data. They developed a platform to extract data from the OLAP cubes stored in the central data warehouse (DW), create ad-hoc Excel reports, and share them through SharePoint.

These operational review meetings proved beneficial and thus continued after the pandemic. The habit of reviewing short-term, operational performance became critical for continuous improvement and thus was “routinized” in MC processes with the label of System for Management (SfM). Today, SfM is a core component of the LRH planning process, with operational meetings taking place every working day at three organizational levels (Table 3):

**Table 3:** System for management (facts and figures)

Level	Time	Attendants	N. of KPIs	# of daily meetings
1	8:00 am	Head of clinical ward (lead), Assistant clinician, Clinical specialist, Lead clinician, Nursing services, Social services	23 KPIs	3
2	9:30 am	Operating room coordinator (lead), Bed manager, Clinical specialist, Heads of clinical wards	- (operating room schedule)	1
	10:00 am	Nursing coordinator (lead), Heads of clinical wards, Sector heads	185 KPIs	3
	12:00 am	CEO (lead), Bed manager, Finance and control, Head of quality, Lead clinicians, Medical Director, Sector heads, Secretariat, Technologist (Radiology)	200 KPIs	1
3	12.15 am	Operating room coordinator (lead), Bed manager, Finance and control, Head of Quality, Lead surgeon, Medical director, Operating room head, Sector heads, Head of Nurses	111 KPIs	1
	2:30 pm	Bed manager and Operating room coordinator (lead), Finance and Control, Lead Clinicians, Medical Director, Domestic Services	43 KPIs	1 (if needed)

1. level 1 (clinical departments);
2. level 2 (inpatient services, outpatient services, operating rooms);
3. level 3 (hospital).

Table 3 summarizes the structure of the SfM operational review cycle, detailing the timing, participants, and scope of performance indicators monitored at each organizational level. It shows how the review process is organized as a multi-level, tightly scheduled sequence of short meetings, with different managerial and clinical actors focusing on distinct operational domains and sets of Key Performance Indicators (KPIs).

The SfM has transformed how we govern the hospital: instead of reacting to problems, we now identify critical signals in real time. [EOC representative #12]

As a rule, all the SfM operational review meetings are short (less than 30 min), highly focused on “red” (underperforming) KPIs, action-oriented, and based on a clear accountability for follow-up (Kaplan and Norton, 2008).

The SfM is now the cornerstone of our daily planning cycle: it keeps track of our operational performance in real time, enables immediate corrective interventions, and fosters a performance-driven culture throughout the whole organization. … The system progressively incorporated an increasing range of KPIs, including quality measures (e.g. average days between patient discharge and the sending of the discharge letter), efficiency measures (e.g. occupancy rates). [EOC representative #1]

SfM is a key process in the LRH planning cycle, and the controlling team has always played a pivotal role in both its development and functioning. Up to late 2023, LRH controllers were in charge of: (1) extracting operational data (manually) from OLAP cubes and other sources; (2) adjusting them with the inputs directly collected from frontline units such as quality management, bed management, etc.; (3) validating data quality and consistency; (4) updating the daily Excel reports, and (5) publishing them on SharePoint before each meeting.

Over time, this information-provider role grew more and more demanding for the LRH controlling team, which had to prepare over 200 meetings every month, with little time left for analyzing data and giving valuable insights into operational performance. Meanwhile, the tendency to broaden the data set for the review meetings jeopardized the effectiveness of SfM. The data quality deteriorated because of the increasing number of inconsistencies across multiple (non-integrated) data sources, delays in data collection, inaccuracies in data validation, etc. These issues undermined the reliability and timeliness of the information provided. At the same time, the leaders of SfM meetings (e.g. heads of clinical departments, bed manager, operating room manager) struggled to “navigate” an overwhelming data set without guidance. Consequently, the dynamics of operational review meetings became somewhat “ritualistic” with discussions becoming trivial and decisions being delayed or diluted.

Over time, the meetings became increasingly ritualistic: discussions often circled around the same issues for weeks, with participants losing focus and energy, and decisions being repeatedly delayed or diluted [EOC representative #7].

A related challenge concerned the sustainability of the improvement logic embedded in SfM. Over time, participants noted a tendency for meetings to shift from action-oriented discussions toward justification rituals, where participants accounted for underperformance rather than collectively identifying corrective responses.

People feel they have to justify themselves. They explain why they didn't reach the target rather than working out how to improve [EOC representative #7].

A further structural limitation concerned the traceability of actions. Throughout the observation period, corrective actions were tracked informally through visual markers within the reporting tool, but no systematic record of completed or abandoned actions was maintained.

Unfortunately, the actions were gradually deleted and there is no trace left [EOC representative #2].

Full accountability therefore depended on individual memory and continuity of participation rather than on the system itself.

In 2024, the hospital's top management launched an initiative to reverse this trend, re-design the informational backbone of SfM and prompt the digital transformation of operational review meetings. Considerable efforts were made to develop the BI platform to support SfM. The extensive use of ETL applications enabled a partial automation of the SfM processes, addressing most data quality issues and smoothing the way for integrating operational data with new (both internal and external) sources. This relieved the LRH controlling team of the burdensome task of collecting and assembling data before the start of each SfM meeting and enabled more flexible, granular, and scalable analyses. The migration from the applications currently in use (Excel, SharePoint) to PowerBI is planned by the end of 2026, when SfM processes will become fully automated.

To date, SfM reports are still manually assembled and distributed by means of standard applications such as Excel and SharePoint. Despite of an old-fashioned frontend, however, the operational data that supports SfM review meetings is now automatically collected and stored in the hospital DW, with no need for further manual work (e.g. cleanings, consolidations, reconciliations, validations). Our goal is now to fully automate the operational review meetings by transitioning to Power BI, thus making operational data accessible to SfM users through a set of dynamic and tailored dashboards to meet the specific needs of levels 1, 2, and 3. [EOC representative #2]

The advancing automation of operational review meetings is facilitating a radical shift in the role of LRH controllers. Rather than acting solely as passive data handlers, LRH controllers are now expected to take the lead on continuous performative improvements in SfM meetings. By automating the “dirty work” of operational data collection, the LRH controlling team is gradually taking an (unprecedented) active role in the SfM meetings. As often as not, controllers are supposed to use their time in operational review meetings to provide not only information, but more importantly, guidance: in how to interpret performance data, identify areas of improvement, and facilitate highly-focused, action-oriented discussions. Given the far-reaching impact of SfM processes, LRH controllers' new role (frequently labeled as “performance leader”) is increasingly recognized by a wide range of internal stakeholders (e.g. top managers, clinical leaders, middle managers), but demands the development of a mixed set of digital (e.g. data governance, ETL applications, data visualization) and soft skills (e.g. communication, relationship management, critical thinking).

With these tools we have a chance of carrying out analyses of a new kind. We no longer confine ourselves to producing figures, but we can now navigate through data and get deep into the weeds. Of course, it is a joint effort with clinicians and managers. [EOC representative #10]

### 4.4 Leveraging predictive analytics for managing risk and uncertainty

As a consequence of the pandemic and its severe impact on the organization's financial performance, pressure to manage operating costs while maintaining investment capacity intensified significantly in early 2024. This was the catalyst for the controlling team to innovate the existing operational planning system and introduce a dynamic, data-driven, and forward-looking forecast.

Thus far, the LRH's operational planning (labeled as *Strategy & Operations Planning* – S&OP) was primarily organized around an annual budget developed between August and November and based upon: (1) EOC's strategic goals and (2) the outcomes of the negotiations with clinical and administrative departments. Once approved (in December), the annual budget served as the sole benchmark for performance evaluation (on a quarterly basis) with no further adjustments. Under this system, the possible shifts in the environmental conditions (e.g. demand for human resources) were not reflected in the annual budget and the performance monitoring remained retrospective.

The HR and ICT Development departments jointly developed a new digital platform to support a forward-looking blueprint for the S&OP processes. The platform was based on: (1) OLAP cubes gathering the relevant HR-related data (previously siloed across several departments such as HR, Finance and Controlling, and Nursing); (2) the monthly financial closings enabled by SAP; (3) the extended array of operational data recently connected to BI applications, and (4) a basic, algorithmic forecast embedded in Excel files.

The S&OP cycle is now performed monthly, which enables the controlling team to track the demand for human and technical resources and to dynamically forecast the hospital's income statement. Forecasts now draw on the improvement initiatives developed under the SfM system, the progress of departmental projects, the estimated trends in volumes, the possible constraints in the hospital's supplied capacity, and the impact of external factors. Under this dynamic system, financial projections are derived monthly from a predictive model (based on multiple variables) whose accuracy is measured as the deviation between actual and forecasted values.

Our operations planning has shifted from a static, retrospective budget logic to a dynamic and integrated S&OP cycle that continuously aligns demand, capacity, and costs, enabling us to anticipate deviations early and adjust resources before financial and operational imbalances materialize. [EOC representative #9]

So far, the digitalization of the S&OP processes has paved the way for the development of data-driven, forward-looking financial forecasts, which have provided the LRH with an early warning system for potential risks and uncertainties emerging from the external environment. Initially triggered by internal financial pressures, the new forecasting model has proven essential for coping with the instability of a fast-changing environment and thus gained a prominent role in LRH control processes.

There was a time when we could set a five-year budget and feel fairly certain it would hold. But today we live in a VUCA world where volatility and uncertainty are part of everyday real life. That is why we need systems that help us to act and not just to predict. We cannot afford to wait and see anymore. [EOC representative #13]

Currently, the model is undergoing further developments meant for: (1) the gradual adoption of advanced predictive analytics, as soon as the implementation of SAP Analytic Cloud from 2026 onward enables the shift toward adaptive forecasting algorithms powered by ML and AI; (2) the increasing use of external data sources (such as demographic trends, epidemiological patterns, and weather forecasts) to enhance the accuracy of S&OP processes; (3) the progressive enrichment of the information ecosystem that powers the dynamic forecast (e.g. the introduction of RFID tags for tracking waste and the use of tablets for monitoring cleaning processes).

We strive for developing an increasingly “predictive” system, something enabling us to make adjustments along the way rather than just at the end of the journey. [EOC representative #1]

Though still in progress, the use of predictive analytics is reshaping LRH controllers' role. As S&OP owners, the controlling team has to perform a new set of tasks (e.g. trend analysis, risk mitigation and response strategies, scenario planning, sensitivity analysis) that goes beyond the simple reporting of financial performance. Increasingly, controllers are expected to assess the possible barriers and/or risks to strategy execution, translate forecasts into actionable insights, and promote strategic alignment under uncertainty. In order to fulfill this new role, the controlling team needs to develop multi-faceted competencies in different domains such as strategic planning, risk management, and advanced statistical techniques. Digitalization did not transform all domains of MC uniformly. While performance visualization, operational monitoring, and forecasting underwent substantial reconfiguration, other areas such as routine accounting, compliance, and parts of budgeting remained relatively stable.

At the end of the day, we need a light for navigating in the mist … this is exactly what we demand from our controllers. [EOC representative #15]

To produce an accurate forecast, you need to understand and manage data from multiple (both internal and external) sources. Without statistical and modeling skills, you’re stuck with rough estimates. [EOC representative #2]

A further domain that remained largely outside the reach of digitalization was the integration of clinical outcome data into MC processes. Performance information continued to be drawn predominantly from financial and operational sources, while quality indicators and clinical outcomes (though available in fragmented form across departmental registers) were not yet systematically incorporated into the control infrastructure.

The thing that is really missing is quality data, for example infection rates, complaints, clinical outcomes. Without that, the picture is incomplete. [EOC representative #13]

This boundary between managerial and clinical information domains represented one of the most significant unresolved tensions in the digitalization trajectory observed.

A related constraint concerned the uneven distribution of technical capabilities within the controlling function itself. The development and maintenance of forecasting tools required competencies in data integration, BI applications, and statistical modelling that were concentrated in a small number of individuals and partially dependent on the ICT Development team. This created a structural vulnerability: the sustainability of the S&OP cycle relied on the continuity of specific expertise rather than on institutionalized organizational capacity, limiting the scalability of the forecasting model beyond its current configuration.

Across the three use cases, digitalization was activated by concrete operational and organizational triggers which destabilized existing control routines and initiated localized trajectories of transformation. Taken together, the three use cases reveal distinct but interrelated digitalization patterns corresponding to visualization-based alignment, automation-driven operational control, and predictive forward-looking planning. These role changes emerged as a consequence of the progressive reconfiguration of control activities rather than as a direct effect of technology adoption itself.

## 5. Discussion

Table 4 synthesizes the three digitalization patterns and makes visible the recurring structure linking triggers, reshaped control activities, technological configurations, and role adjustments. Building on this empirical synthesis, the following discussion develops a processual interpretation of digitalization as a patterned reconfiguration of MC and PM activities over time.

**Table 4:** Mechanisms and trajectories of digitalization

Dimension of analysis/Use case	Leveraging data visualization tools for creating strategic alignment	Leveraging business intelligence and analytics for automating operational review meetings	Leveraging predictive analytics for managing risk and uncertainty
Trigger	Need for shared, visible performance information; lack of integrated view; coordination and accountability pressures	Manual data inefficiencies; need for real-time operational monitoring; reporting burden	Rising uncertainty and cost pressure; need to anticipate resources and capacity; limits of retrospective control
Digital technologies adopted	BI platforms; interactive dashboards; integrated visualization tools	Automated data extraction; multi-source integration; BI-based operational monitoring; workflow automation	Advanced analytics; predictive forecasting tools; integration of operational and financial data
Controller activities impacted	Performance visibility and alignment; cross-level coordination; benchmarking and monitoring	Operational control; data collection and reporting; real-time performance reviews	Planning and forecasting; risk management; forward-looking performance management
Nature of change in control activities	From fragmented, retrospective reporting → integrated, multidimensional visualization; enhanced transparency and shared interpretive frame	From manual data preparation → automated, routinized monitoring; increased frequency and granularity of control	From retrospective control → predictive and anticipatory control; enhanced scenario-based decision support
Extent of transformation	Selective: strong impact on visibility and coordination; limited transformation of core financial control	Selective: deep transformation of operational control; relative stability of strategic and financial control	Selective but deep: major transformation of planning and forecasting; limited impact on routine accounting and compliance
Change in controller's role	From data consolidator → interpreter and facilitator of performance dialogue; stronger coordination role	From data handler → performance process owner; responsibility for data reliability and operational monitoring	From scorekeeper → forward-looking analyst and decision-support partner; stronger role in scenario evaluation and uncertainty management
Professional roles involved	Controllers; operational managers; clinical/functional leaders; IT/BI specialists	Controllers; operations managers; IT and data teams; process owners	Controllers; top management; planning and finance teams; data analysts; IT specialists
Emerging tensions and challenges	Data ownership and interpretation; transparency vs information overload	Data governance and quality; coordination with ICT; integration complexity	Forecast reliability; analytical capability; managing uncertainty and model dependence
Contribution to digitalization trajectory	Enables data-driven coordination and strategic alignment	Establishes routinized, automated operational control infrastructure	Extends control toward predictive, anticipatory, and risk-oriented management

The first research question examines how digitalization reshapes MC and PM activities over time. While prior literature has extensively discussed the transformative potential of digital technologies for MC, most studies focus on individual tools or isolated control tasks, providing limited insight into how everyday control activities evolve in practice (Möller *et al.*, 2020; Fähndrich, 2023; Papiorek and Hiebl, 2024; Martins *et al.*, 2025). In healthcare, performance information operates as a core coordination and governance mechanism supporting accountability, organizational learning, and performance improvement across multiple levels (Buttigieg *et al.*, 2017; Mauro *et al.*, 2024). However, existing research rarely explains how the production, integration, and use of performance information change as digitalization unfolds.

The findings show that digitalization reshapes MC primarily through the transformation of control activities rather than through the mere introduction of new technologies. Across the case, three interrelated dimensions of change emerge: the temporal orientation of control, the integration and depth of performance information, and the practical use of control outputs in decision-making and coordination.

First, digitalization transformed the temporal orientation of control. Control progressively shifted from retrospective and periodic evaluation toward real-time monitoring and, ultimately, toward forward-looking performance management. Initially, performance information was produced at long intervals and used mainly for ex post review. As digital infrastructures developed, performance data became accessible in near real time, enabling faster monitoring and response. The integration of predictive analytics further extended control toward anticipatory decision support and proactive management of uncertainty.

Second, digitalization deepened informational integration. Prior to digital transformation, performance information was fragmented across financial, operational, and clinical domains, limiting its usefulness for cross-level coordination. The progressive integration of data sources enabled multidimensional and more reliable performance information, strengthening transparency and shared interpretation across organizational and professional boundaries, and reinforcing the coordinating function of PM systems in healthcare (Buttigieg *et al.*, 2017; Mauro *et al.*, 2024).

Third, digitalization reshaped how control outputs are used in practice. Control evolved from a reporting-oriented function toward an interpretive and decision-support mechanism embedded in ongoing organizational processes. Automation reduced time devoted to manual data handling and increased focus on interpretation and coordination, while predictive capabilities strengthened the role of control in guiding forward-looking decisions under uncertainty. Beyond changes in timing and use, digitalization transforms the systemic function of performance measurement. PMS progressively evolve from evaluative infrastructures focused on ex post measurement, to coordination mechanisms enabling real-time alignment, and ultimately toward anticipatory systems supporting forward-looking governance under uncertainty.

Importantly, this transformation remained selective and uneven across control domains. While performance visualization, operational monitoring, and forecasting underwent substantial reconfiguration, other areas such as routine accounting, compliance, and parts of budgeting remained relatively stable. This selective evolution explains why digitalization produces heterogeneous outcomes and supports a view of digital transformation as a situated and progressive reconfiguration of control activities rather than a uniform technological shift. At the same time, the case reveals an inherent structural tension within digitally transforming control systems. Digitalization simultaneously stabilizes control by improving data integration, automation, and timeliness, and destabilizes it by increasing interpretive demands, temporal pressure, and reliance on complex information infrastructures. This tension resonates with Quattrone's (2016) warning that the move to digital does not automatically produce wiser decision-making. As he argues, increasing data availability risks reducing accounting to a consumption activity, numbers packaged and made available for use without being questioned or debated, rather than a communicative practice that interrogates organizational realities. The empirical evidence presented here supports this concern: expanded data visibility did not automatically translate into data literacy, quality indicators remained outside the reach of the control infrastructure, and the interpretive demands placed on controllers grew even as the volume of available information increased.

As a result, control becomes more powerful in coordinating and anticipating organizational dynamics, yet more exposed to coordination fragility, data dependency, and the risk of interpretive overload.

The second research question examines what triggers and mechanisms explain the emergence of different digitalization trajectories in MC and PM. Prior literature recognizes digitalization as a context-dependent and non-linear phenomenon shaped by technological, organizational, and environmental conditions, yet often remains vague about how transformation is activated and unfolds in practice (Korhonen *et al.*, 2021; Oesterreich *et al.*, 2019; Begkos *et al.*, 2024). Empirical evidence on concrete triggers and generative mechanisms remains limited, particularly in complex professional settings such as healthcare (Kjekshus and Bygstad, 2024).

The findings show that digitalization is not primarily driven by a single, deliberate digital strategy but is activated by specific contextual pressures that expose the limits of existing control practices. In this study, triggers are conceptualized not as isolated events but as underlying pressures revealing a misfit between existing control practices and evolving organizational demands. These pressures expose the limits of established routines and activate distinct digitalization trajectories rather than initiating a uniform or centrally planned transformation. These triggers destabilize established routines and generate adaptive responses that progressively reshape control activities. Three distinct triggers emerged.

An informational trigger, intensified during the COVID period, created an urgent need for timely and accessible performance information to support coordination and visibility. This pressure led to the adoption of visualization tools, which progressively transformed PM by enabling shared interpretation and real-time access to multidimensional data.

An operational trigger emerged from the growing burden of manual data handling and declining data reliability in operational review processes. The introduction of BI infrastructure and automated data integration stabilized information flows and enabled routinized, scalable performance monitoring, shifting control toward more structured and action-oriented practices.

A financial and strategic trigger arose from increasing uncertainty and cost pressure, revealing the limits of retrospective control. The adoption of predictive analytics enabled forward-looking assessment of performance and resource use, extending control toward anticipatory and risk-oriented decision support.

Across the case, these trajectories were not synchronized but unfolded in a staggered and path-dependent manner, each evolving at different speeds and affecting different control domains. This explains why digitalization does not appear as a single, organization-wide transformation but as a configuration of coexisting and heterogeneous patterns. By identifying how specific triggers activate distinct transformation paths, the study clarifies the mechanisms through which digitalization unfolds and explains the uneven and context-dependent nature of digital transformation highlighted in prior literature (Korhonen *et al.*, 2021; Fähndrich, 2023; Begkos *et al.*, 2024).

Taken together, the findings show that the digitalization of MC does not unfold as a uniform transformation of systems but as a patterned reconfiguration of control activities over time. Across the case, a recurring process logic emerges in which contextual triggers activate distinct digitalization patterns, these patterns reshape how performance information is produced and used, and the controller's role evolves as a cumulative outcome of these activity-level transformations. This perspective shifts attention from technologies or systems *per se* to the micro-level mechanisms through which digitalization becomes consequential for control work (Möller *et al.*, 2020; Fähndrich, 2023; Begkos *et al.*, 2024).

The empirical evidence can be synthesized into a process model linking four interrelated stages: trigger, pattern activation, activity reshaping, and role reconfiguration. First, concrete organizational pressures reveal the limits of existing control routines. Second, these pressures activate specific digitalization patterns associated with different technological and organizational configurations. Third, these patterns progressively reshape the content, integration, and temporal orientation of control activities, moving from retrospective reporting toward real-time monitoring and forward-looking anticipation. Finally, role reconfiguration emerges indirectly as controllers adapt to the evolving demands of digitally mediated control work.

Digitalization becomes organizationally transformative only when the reshaping of control activities alters the temporal and interpretive structure of performance information use. Transformation therefore depends not merely on technology adoption but on the stabilization of new ways in which performance information is produced, mobilized, and enacted within recurring organizational processes. When such stabilization does not occur, digitalization may remain technological without producing substantive change in control.

This mechanism explains why digitalization is partial, uneven, and heterogeneous across control domains. The effectiveness of this mechanism depends on specific boundary conditions, including data integration capacity, cross-functional coordination, and analytical competencies within the controlling function. When these conditions are weak or absent, digitalization may remain technological without becoming organizationally transformative. These boundary conditions are not abstract prerequisites but emerged concretely across the three use cases. Data integration capacity proved critical in the SfM case, where the absence of automated data flows initially undermined the reliability and timeliness of operational reviews. Cross-functional coordination was essential in the visualization case, where the controlling team had to actively bridge clinical and administrative domains to make performance dashboards meaningful and actionable. Analytical competencies emerged as a constraining factor in the forecasting case, where the shift toward predictive analytics required controllers to develop statistical and modelling skills that were not part of their traditional professional repertoire. Rather than a single transformation, multiple trajectories coexist, evolve at different speeds, and produce different effects depending on triggers, organizational conditions, and the stabilization of digitally mediated control practices (Korhonen *et al.*, 2021; Kjekshus and Bygstad, 2024). By making these patterned trajectories visible, the study advances the literature on MC digitalization by providing a processual explanation of how digital transformation unfolds in practice and why its outcomes vary across contexts (Fähndrich and Pedell, 2025; Begkos *et al.*, 2024).

This study offers four interrelated contributions to the literature on MC digitalization, PM in healthcare, and the evolving role of controllers.

First, the study introduces a pattern-based conceptualization of MC digitalization. Rather than interpreting digitalization as the adoption of discrete technologies or as a uniform organizational shift, the findings show that digitalization unfolds through distinct but recurring patterns, each reshaping specific clusters of control activities. This perspective shifts the analytical focus from technologies themselves to how digital tools progressively reconfigure control work overtime (Möller *et al.*, 2020; Fähndrich and Pedell, 2025).

Second, the study advances processual research by identifying a concrete mechanism through which digitalization operates. The evidence shows that digital transformation proceeds through a sequence in which contextual triggers activate digitalization patterns, patterns reshape control activities, and activity changes gradually reconfigure professional roles. Digitalization therefore emerges as an ongoing process of cumulative adjustments in how control work is performed, extending prior processual accounts of digital transformation (Begkos *et al.*, 2024).

Third, the study contributes to the literature on PM and MC in healthcare by showing how digitalization reshapes the nature and use of performance information. The findings reveal a progressive shift from retrospective reporting toward real-time monitoring and forward-looking control. Along this trajectory, performance information evolves from a reporting device to a coordination mechanism and ultimately to an anticipatory tool supporting decision-making under uncertainty, thus adding a dynamic and temporal perspective to existing research on healthcare performance systems.

Fourth, the study reframes the evolving role of controllers as an emergent outcome of digitally mediated transformations of control activities. Rather than pushing controllers toward a predetermined trajectory, digitalization generates multiple and coexisting role configurations depending on which activities are reshaped and how transformation unfolds over time. Role change is therefore not driven directly by digital technologies but emerges from the reconfiguration of control work they enable, positioning role transformation as a cumulative outcome of evolving control activities rather than as a predefined “digital role”. This perspective helps reconcile prior mixed evidence in the literature and highlights the contingent and processual nature of role change in digitally transforming organizations (Oesterreich *et al.*, 2019; Rautiainen *et al.*, 2024; van Slooten *et al.*, 2024).

The patterns identified in this study should not be interpreted as descriptive categorizations but as generative and analytically generalizable configurations explaining how digitalization becomes consequential for control work. Positioned within a processual and path-dependent perspective, these patterns capture recurring mechanisms through which digital transformation unfolds across control domains.

## 6. Conclusions

This paper examines how the digitalization of MC unfolds in a complex healthcare organization through a longitudinal, interventionist study conducted in a Swiss multisite public hospital. Rather than treating digitalization as the adoption of discrete technologies, the study conceptualizes it as a progressive reconfiguration of control activities that reshapes how performance information is produced, integrated, and used within organizational processes. By following the transformation over time, the analysis reveals how digitalization becomes consequential for everyday control work and for the functioning of PM as a core governance infrastructure in healthcare. While empirically grounded in a healthcare setting, the mechanism identified in this study is not sector-specific but relates to the nature of control work under digitalization. The patterned interaction between triggers, activity reshaping, and role evolution provides analytically generalizable insights into how digital transformation unfolds in complex, data-intensive organizational contexts.

Taken together, the findings provide a coherent answer to the research questions guiding this study. First, digitalization progressively reshapes MC and PM activities by transforming the production, integration, and mobilization of performance information, shifting control from retrospective evaluation toward increasingly real time and forward-looking coordination. Second, the study identifies contingent triggers and generative mechanisms that explain why digitalization unfolds through heterogeneous and sometimes coexisting trajectories rather than along a linear path. Third, the findings show that role transformation does not stem directly from technology adoption but emerges through the reconfiguration of control activities, leading to evolving and hybrid configurations of the controller's role over time.

This study adopts a processual and practice-based perspective on the digital transformation of management control. Rather than viewing digitalization as a discrete technological shift, the analysis explains how evolving control practices, organizational conditions, and professional roles interact over time to produce situated transformation trajectories. In doing so, the study moves beyond technology centric explanations and contributes to the literature by showing that digitalization becomes consequential when it reshapes the concrete activities through which performance information is generated, integrated, and used. This perspective helps clarify why digital transformation produces uneven outcomes across control domains and why its organizational implications vary across contexts.

The findings indicate that digitalization unfolds through the interaction of contingent triggers, evolving control practices, and progressive role reconfiguration. Organizational pressures expose the limits of existing control routines and activate specific digitalization patterns. These patterns progressively reshape the content, integration, and temporal orientation of control activities, and role transformation emerges as a cumulative outcome of these activity level changes. Digital transformation therefore appears as an adaptive and path dependent process rather than a purely technology driven or fully planned shift, explaining the selective, heterogeneous, and evolving nature of digitalization in management control.

This study has some limitations. First, the evidence is drawn from a single interventionist case in a Swiss multi-site hospital, which limits statistical generalizability beyond similar data rich and coordination intensive settings. Second, the interventionist design, while enabling deep and longitudinal access to real-time organizational change, carries inherent limitations that deserve explicit acknowledgement. As active participants in the digitalization process, the researchers were simultaneously agents and observers of change, which may have introduced a prospective bias in how transformation trajectories were framed and interpreted. Although the two academic authors conducted data analysis independently and the organizational co-author did not participate in coding, the collaborative nature of the engagement may have encouraged participants to present digitalization in a favorable light, particularly in the ex-post interviews conducted after the main intervention period. Moreover, the reliance on participants' retrospective accounts of change processes introduces a further risk of narrative selectivity, as informants may emphasize successful outcomes while downplaying tensions, resistances, or failed initiatives. These limitations suggest that the findings should be read as a situated and reflexively constructed account of digitalization rather than as a neutral description of events.

This study opens several avenues for future research. Further work is needed to examine whether similar digitalization patterns emerge in other organizational and sectoral contexts, particularly in environments characterized by lower data maturity or fragmented information infrastructures. Future studies could investigate the temporal interaction and sequencing of multiple digitalization patterns over longer periods, as well as the conditions under which digitalization enhances or fails to enhance the use of performance information for coordination and decision making. Additional research should also explore situations in which digitalization does not lead to significant role reconfiguration, in order to better understand boundary conditions and failed transformations. Finally, as predictive analytics, ML, and AI become increasingly embedded in control processes, future research could examine how these technologies further reshape control activities, temporal orientations, and professional roles in complex and uncertainty laden environments such as healthcare.

From a healthcare management perspective, future research could investigate how digitalization reshapes the relationship between clinical and administrative professionals, particularly in settings where performance information operates at the intersection of managerial and clinical logics. Further work could also examine how different healthcare system configurations, including varying levels of digital maturity, funding models, and regulatory environments, shape the adoption and consequences of MC digitalization, thereby extending the generalizability of the patterns identified here. Finally, given the growing emphasis on integrated care and value-based healthcare, future studies could explore how digitally transformed control systems support or hinder the coordination of care across organizational and professional boundaries, with implications for both healthcare governance and the training of hybrid professionals operating at the interface of clinical and managerial domains.

## Appendix

**Table A1:** List of acronyms and definition of technologies

Acronym	Acronym	Definition of the technology
MC	Management Control	–
MCS	Management Control Systems	–
PMS	Performance Management Systems	–
KPI	Key Performance Indicator	–
BI	Business Intelligence	Digital systems that integrate, process, and visualize data to support monitoring and decision-making through dashboards and analytics
ERP	Enterprise Resource Planning	Integrated information system that manages core organizational processes (e.g. accounting, procurement, HR) through a centralized database
AI	Artificial Intelligence	Computational systems capable of performing tasks requiring human-like cognitive functions such as prediction, pattern recognition, and decision support
ICT	Information and Communication Technology	–
BSC	Balanced Scorecard	–
DRG	Diagnosis-Related Group	–
SfM	System for Management	–
DW	Data Warehouse	Centralized data repository that integrates information from multiple sources to support reporting, analytics, and performance monitoring
ML	Machine Learning	Subfield of AI that enables systems to learn from data and improve predictions or classifications without explicit programming
